# Impact of childhood trauma on cognitive function in patients with bipolar disorder

**DOI:** 10.3389/fpsyt.2025.1513021

**Published:** 2025-01-23

**Authors:** Zhiyang Zhang, Chenyu Zhou, Zhen Mao, Yue Sun, Lei Zhao, Tian Li, Chuanyue Wang, Qijing Bo

**Affiliations:** ^1^ The National Clinical Research Center for Mental Disorders & Beijing Key Laboratory of Mental Disorders & Beijing Institute for Brain Disorders Center of Schizophrenia, Beijing Anding Hospital, Capital Medical University, Beijing, China; ^2^ Advanced Innovation Center for Human Brain Protection, Capital Medical University, Beijing, China

**Keywords:** bipolar disorder, childhood trauma, cognition, neuropsychological assessment, abuse and neglect

## Abstract

**Background:**

Patients with bipolar disorder (BD) frequently exhibit cognitive impairments. However, the association between childhood trauma as a risk factor for BD and cognitive deficits remains ambiguous.

**Objective:**

To investigate the relationship between childhood trauma and cognitive function among patients with BD.

**Methods:**

The study included 90 patients with BD and 94 healthy controls (HC). Childhood trauma was assessed using the Childhood Trauma Questionnaire (CTQ), and cognitive function was evaluated using the Repeatable Battery for the Assessment of Neuropsychological Status (RBANS). The relationships between childhood trauma and cognitive function.

**Results:**

In BD group, childhood abuse and neglect were more prevalent than in HC group. Mood stabilizer use was positively associated with language abilities, while antipsychotic use negatively impacted attention. Emotional abuse predicted impaired immediate memory, with the number of episodes and valproate dosage negatively correlating with total RBANS scores, whereas education and mood stabilizer use showed positive correlations.

**Conclusions:**

The incidence of childhood trauma was higher among BD than HC, and different types of childhood trauma had varying effects on different aspects of cognition. These studies will deepen the understanding of the complexity of BD and support the development of more effective treatment methods.

## 1 Introduction

Bipolar disorder (BD) is a severe, chronic mental disorder marked by variations in mood and energy levels that affects more than 1% of the global population (1). In a 2019 epidemiological investigation of mental disorders in China, the lifetime prevalence of BD was reported to be 0.6% (2). An expanding body of research confirms that BD patients frequently experience persistent and far-reaching cognitive impairments, notably in domains such as attention, language acquisition, memory, and executive function (3). Childhood trauma refers to adverse events that occur in an individual’s life before the age of 18 years. Categories of childhood trauma include childhood abuse (psychological abuse, physical abuse, and sexual contact abuse), neglect (physical neglect and emotional neglect), and exposure to household dysfunction (family members abusing substances, having mental illness, engaging in criminal behavior, or being victims of violent treatment by relatives) (4).

Between 25% and 50% of patients with mental disorders are reported to have experienced childhood trauma (5). A previous meta-analysis found that childhood trauma constitutes a risk factor for the onset of BD (6). BD are 2.6 times more likely to report a history of childhood trauma compared with healthy controls (HC) (7). In recent cross-sectional studies, the prevalence of childhood trauma among BD was found to be 50.2%, with 7.5% reporting physical abuse, 21.9% experiencing neglect, and 20.8% having a history of both abuse and neglect (8). Moreover, research has shown that a background of childhood trauma is associated with both the vulnerability to and the severity of BD (9). BD with lower cognitive functioning are more likely to have experienced childhood trauma (10). Research has also indicated that a certain relationship exists between childhood trauma and cognitive impairments in BD, with studies finding that patients with a history of childhood trauma who experience manic episodes exhibit significant deficits in overall cognitive function, executive function, and attention (11).

Previous studies have shown that childhood trauma (such as emotional abuse, physical neglect, and emotional neglect) significantly impacts the cognitive functions of patients with BD. Emotional abuse notably affects the intellectual development and executive functions of BD. Research indicates that childhood emotional abuse is associated with cognitive impairments, particularly in memory, executive function, and attention (10). Additionally, emotional abuse is linked to an earlier onset of BD, more suicide attempts, and greater emotional instability (12). Physical neglect is associated with impaired executive function and attention in BD. Studies show that physical neglect is linked to cortical dysfunction, which affects patients’ executive function and attention (13). Physical neglect is also related to diminished emotional regulation, leading to higher impulsivity and emotional instability in BD (14). Emotional neglect has a significant impact on cognitive functions, particularly in intelligence and executive function. A previous study indicated that childhood emotional neglect is closely associated with reduced intelligence and impaired executive function. The study also mentioned that physical abuse is negatively correlated with years of education and estimated intelligence, suggesting that physical abuse may further affect intellectual development by impacting educational experiences (15). Furthermore, emotional neglect is associated with increased suicide risk and greater emotional instability in BD (16). In summary, childhood trauma, especially emotional abuse, physical neglect, and emotional neglect, has significant negative effects on the cognitive functions of BD patients, such as reduced intelligence, impaired executive function, decreased attention, and diminished emotional regulation.

While the current literature indicates that childhood trauma likely poses an additional risk to cognitive functioning in patients with BD, prior studies have not thoroughly examined how specific types of childhood trauma affect diverse aspects of cognition in patients with BD. This study aims to explore the association between childhood trauma and cognitive function, contributing to a deeper understanding of the long-term impact of trauma on cognition. By uncovering these mechanisms, the study provides valuable insights for the early identification of cognitive impairments and the development of personalized therapeutic strategies. Additionally, investigating the influence of factors such as educational level, number of episodes, and medication on cognitive function can guide comprehensive management across the entire course of the illness. This approach seeks to improve patients’ quality of life and social functioning. Ultimately, this study may facilitate a shift from traditional symptom management to holistic functional enhancement, offering more comprehensive care and support for BD.

## 2 Methods

### 2.1 Study participants

The study included patients with BD in the remission phase or a depressive episode who were receiving outpatient or inpatient care at Beijing Anding Hospital, Capital Medical University. A convenient sampling method was employed, with a total of 90 patients included. The inclusion criteria were as follows: 1) meeting the diagnostic criteria for BD as defined in the Fourth Edition of the Diagnostic and Statistical Manual of Mental Disorders (DSM-IV); 2) having completed at least 9 years of education, with a reasonable level of Chinese reading and comprehension ability; 3) aged between 18 and 55 years; 4) not having received modified electroconvulsive therapy within the previous 3 months; and 5) The following exclusion criteria were applied: 1) history of alcohol and substance abuse, intellectual disability, dementia, traumatic brain injury, or any severe chronic physical illness; 2) presentation of severe self-harm, suicidal thoughts, or ideation during the study period; 3) pregnancy or lactation; 4) total Young Mania Rating Scale (YMRS) score >5 points; and 5) a definite diagnosis of any mental disorder other than BD. Additionally, 94 HC were included in the study. The inclusion criteria for HC were as follows: 1) not meeting any diagnostic criteria for mental disorders as defined in the Fourth Edition of the Diagnostic and Statistical Manual of Mental Disorders (DSM-IV); the other inclusion criteria were the same as for the BD group. Exclusion criteria included: 1) participants currently taking antidepressants, anxiolytics, or other psychotropic medications; the other criteria were the same as for the BD group. Approval of this study was granted by the Ethics Committee of Beijing Anding Hospital, Capital Medical University, with ethics reference number: (2013) Research No. (10). All participants willingly joined the study and provided written informed consent, with their guardians’ approval.

### 2.2 Assessments

The 17-item Hamilton Depression (HAMD-17) scale (17) was used to assess the severity of depressive symptoms in patients. The HAMD-17 is currently the most commonly used scale internationally for evaluating the severity of depressive symptoms, offering good reliability and validity. The scale consists of 17 items, each scored from 0 to 4 or 0 to 2, with higher scores indicating more severe depression.

The Young Mania Rating Scale (YMRS) (18) was used to assess the severity of manic symptoms in participants. The YMRS is the most commonly used clinician-rated scale for assessing manic symptoms. The scale includes 11 items, with items 1, 2, 3, 4, 7, 10, and 11 scored from 0 to 4, while the other items are scored from 0 to 8, with higher scores indicating more severe manic symptoms.

The Childhood Trauma Questionnaire (CTQ) (19) was developed by American psychologist Bernstein and colleagues and is used to assess childhood trauma experiences with good reliability and validity. The Chinese version of the CTQ has good reliability and validity, as confirmed by researchers such as Zhang Min. (20). The questionnaire consists of five subscales, including emotional abuse, physical abuse, sexual abuse, emotional neglect, and physical neglect, with a total of 28 items. Higher scores indicate more severe childhood trauma. The presence of childhood trauma is defined as meeting any of the following conditions: emotional abuse score ≥ 13, physical abuse score ≥ 10, sexual abuse score ≥ 8, emotional neglect score ≥ 15, and physical neglect score ≥ 10.

The Repeatable Battery for the Assessment of Neuropsychological Status (RBANS) (21) was used to assess cognitive function. The RBANS is a comprehensive cognitive assessment tool that has been validated in multiple countries, including China. The Chinese version of the RBANS has good reliability and validity, as confirmed by researchers such as Zhang et al. (22). The RBANS includes five dimensions: immediate memory (story recall, list learning), visual span (line orientation, graphic reproduction), language (semantic fluency, picture naming), attention (digit span, coding test), and delayed memory (story retelling, list recall, figure recall, list recognition). There are 12 subtests in total. The raw scores for each dimension are converted into total scores by referring to tables.

### 2.3 Data collection

This study was conducted by trained psychiatrists. They provided participants and their guardians with a comprehensive explanation of the study’s background, objectives, content, significance, and the use of questionnaires. Informed consent was obtained before collecting general demographic and clinical data from the participants. Scale assessments were performed by psychiatric raters who had undergone uniform training and completed when participants could provide sufficient time and cooperation.

### 2.4 Statistical methods

Data analysis was performed using SPSS Statistics v26.0 for Windows (IBM Corp., Armonk, NY, USA). Descriptive statistical analysis was conducted on participants’ age, marital status, years of education, family history, age at first onset, total duration of illness, number of episodes, HAMD-17 total score, YMRS total score, and the incidence of childhood trauma. For normally distributed continuous data, the mean and standard deviation (± SD) were used for descriptive statistics, while for non-normally distributed data, the median and interquartile range [M (P25, P75)] were applied. Categorical variables were analyzed using chi-square tests, and the results were reported as frequencies or percentages (%). We then used descriptive statistical analysis to examine the differences in cognitive function between BD patients with at least one type of abuse and those without any abuse, as well as between the BD and HC groups. Considering the impact of medication on cognitive function, we analyzed the use of mood stabilizers, antipsychotics, and antidepressants, as well as the effects of medication dosages on cognition (antipsychotic dosages were converted to olanzapine equivalents, and antidepressant dosages were converted to fluoxetine equivalents). Spearman correlation analysis was used to evaluate the relationships between RBANS scores and general factors such as gender and age, as well as CTQ scores. Subsequently, multivariate regression analysis was conducted to further explore the relationship between participants’ CTQ scores and RBANS scores. In the multivariate regression analysis, the five dimensions of the CTQ were used as independent variables, with gender, age, and years of education as covariates, and the five dimensions of the RBANS were treated as dependent variables. Differences with two-tailed P < 0.05 were considered statistically significant. Finally, general linear regression analysis was applied to assess the relationships between CTQ total score, age, gender, and RBANS total score.

## 3 Results

### 3.1 General demographic and clinical characteristics as well as rates of childhood trauma

The data for gender, age, marital status, years of education, family history, age at first onset, total duration of illness, number of episodes, HAMD-17 total score, YMRS total score, and incidence of childhood trauma in both groups are presented in **Table 1**. The incidence rates of childhood emotional abuse, physical abuse, sexual abuse, emotional neglect, and physical neglect in the 90 patients with BD were 10%, 8.89%, 17.78%, 24.44%, and 44.44%, respectively. In the group of 94 HC, these incidence rates were 0.00%, 2.13%, 5.32%, 8.51%, and 32.98%, respectively.

**Table 1:** Demographic data, clinical data, and childhood trauma occurrence rates among patients with bipolar disorder (BD) and healthy controls (HC).

Items	BD group (n=90)	HC group (n=94)	Test statistics	*P*-value
Male [n (%)]	56 (62.22)	42 (44.68)	5.683 (x^2^)	0.017
Age (year, mean ± SD)	30.26 ± 10.26	33.61 ± 10.47	-1.801 (Z)	0.072
Married [n (%)]	36 (40.00)	47 (50.00)	1.857 (x^2^)	0.173
Years of education (years, $\bar{\text{x}}$ )	13.27 ± 3.17	14.21 ± 3.00	-2.168 (Z)	0.030
Positive family history of BD [cases (%)]	27 (30.00)	\	\	\
Age at first onset [year, M (P_25_, P_75_)]	22.00 (17.00, 28.00)	\	\	\
Total duration of BD [month, M (P_25_, P_75_)]	61.00 (24.00, 96.00)	\	\	\
Number of episodes [times, M (P_25_, P_75_)]	3.00 (2.00, 4.00)	\	\	\
HAMD-17 total score [points, M (P_25_, P_75_)]	4.00 (1.00, 11.00)	0.00 (0.00, 0.00)	-9.144 (Z)	< 0.001
YMRS total score [points, M (P_25_, P_75_)]	1.00 (0.00, 4.00)	0.00 (0.00, 1.00)	-3.609 (Z)	< 0.001
Emotional abuse [n (%)]	9 (10.00)	0 (0.00)	3.14 (Z)	0.001
Emotional abuse score [points, M (P_25_, P_75_)]	6,11 (5.00, 7.00)	7.71 (5.00, 9.00)	9.883 (x^2^)	0.002
Physical abuse [n (%)]	8 (8.89)	2 (2.13)	2.02 (Z)	0.043
Physical abuse score [points, M (P_25_, P_75_)]	6.16 (5.00, 5.00)	5.68 (5.00, 5.00)	4.090 (x^2^)	0.043
Sexual abuse [n (%)]	16 (17.78)	5 (5.32)	2.66 (Z)	0.007
Sexual abuse score [points, M (P_25_, P_75_)]	6.20 (5.00, 6.00)	5.41 (5.00, 5.00)	7.059 (x^2^)	0.008
Emotional neglect [n (%)]	22 (24.44)	8 (8.51)	2.92 (Z)	0.003
Emotional neglect score [points, M (P_25_, P_75_)]	11.88 (9.00, 14.25)	9.77 (7.00, 11.00)	8.554 (x^2^)	0.003
Physical neglect [n (%)]	35 (44.44)	31 (32.98)	0.84 (Z)	0.403
Physical neglect score [points, M (P_25_, P_75_)]	9.14 (6.00, 11.25)	8.00 (5.00, 10.00)	0.698 (x^2^)	0.403

BD, bipolar disorder; HC, healthy control; HAMD-17, The 17-item Hamilton Depression scale; YMRS, Young Mania Rating Scale.

### 3.2 Comparison of cognitive function across different groups

Participants with any of the following Childhood Trauma Questionnaire (CTQ) scores—emotional abuse ≥ 13, physical abuse ≥ 10, sexual abuse ≥ 8, emotional neglect ≥ 15, or physical neglect ≥ 10—were classified as having experienced childhood trauma. We compared RBANS scores between BD patients with childhood trauma and those without. As shown in **Table 2**, no significant differences were found between these two groups. **Table 3** presents the RBANS scores for the BD and HC groups. The HC group scored higher than the BD group in total RBANS scores, as well as in dimensions including immediate memory, list learning, story recall, line orientation, language function, semantic fluency, attention, coding test, delayed memory, story retelling, list recall, list recognition, and figure recall (all P < 0.05).

**Table 2:** Comparison of RBANS between BD with childhood trauma and without childhood trauma.

Items	BD With Childhood Trauma (n=50)	BD Without Childhood Trauma (n=40)	*P*-value
RBANS total score [M (P_25_, P_75_)]	85.0 (80.0, 93.0)	86.0 (80.5, 95.5)	0.429
Immediate memory [M (P_25_, P_75_)]	83.0 (69.0, 90.0)	83.0 (73.0, 90.0)	0.517
List learning [M (P_25_, P_75_)]	27.0 (22.0, 30.0)	28.0 (23.0, 30.5)	0.530
Story recall [M (P_25_, P_75_)]	14.0 (11.0, 17.0)	14.0 (13.0, 17.5)	0.311
Visual span [M (P_25_, P_75_)]	100.0 (87.0, 109.0)	100.0 (92.0, 108.5)	0.817
Graphic reproduction [M (P_25_, P_75_)]	20.0 (17.0, 20.0)	20.0 (19.0, 20.0)	0.192
Line orientation [M (P_25_, P_75_)]	17.0 (15.0, 19.0)	17.0 (14.0, 19.0)	0.650
Language function [M (P_25_, P_75_)]	87.0 (78.0, 96.0)	87.0 (78.0, 99.0)	0.900
Picture naming [M (P_25_, P_75_)]	10.0 (9.0, 10.0)	10.0 (9.0, 10.0)	0.478
Semantic fluency [M (P_25_, P_75_)]	17.0 (14.0, 20.0)	18.0 (14.5, 21.0)	0.252
Attention [M (P_25_, P_75_)]	100.0 (91.0, 109.0)	106.0 (91.0, 113.5)	0.308
Digit span [M (P_25_, P_75_)]	15.0 (14.0, 16.0)	16.0 (15.0, 16.0)	0.154
Coding test [M (P_25_, P_75_)]	43.0 (37.0, 50.0)	47.0 (36.5, 57.0)	0.249
Delayed memory [M (P_25_, P_75_)]	85.0 (77.0, 94.0)	85.0 (80.0, 94.0)	0.670
Story retelling [M (P_25_, P_75_)]	8.0 (5.0, 9.0)	8.0 (7.0, 10.0)	0.317
List recall [M (P_25_, P_75_)]	5.0 (4.0, 7.0)	6.0 (5.0, 7.5)	0.157
List recognition [M (P_25_, P_75_)]	20.0 (18.0, 20.0)	20.0 (19.0, 20.0)	0.442
Figure recall [M (P_25_, P_75_)]	12.0 (9.0, 16.0)	13.0 (7.5, 16.0)	0.987

BD, bipolar disorder; RBANS, Repeated Battery for the Assessment of Neuropsychological Status.

**Table 3:** RBANS total score, scores for 5 domains, and scores for 12 subtests in patients with BD and HC.

	BD group (n=90)	HC group (n=94)	*P*-value
RBANS total score (mean ± SD)	86.35 ± 11.66	97.73 ± 14.70	< 0.001
Immediate memory (mean ± SD)	81.40 ± 16.46	91.52 ± 17.95	< 0.001
List learning (mean ± SD)	26.43 ± 6.37	29.71 ± 5.34	< 0.001
Story recall (mean ± SD)	7.58 ± 2.62	8.97 ± 2.96	< 0.001
Visual span (mean ± SD)	97.95 ± 16.08	100.87 ± 17.02	0.167
Graphic reproduction (mean ± SD)	18.93 ± 1.57	18.47 ± 2.40	0.517
Line orientation (mean ± SD)	16.58 ± 3.15	17.60 ± 2.32	0.045
Language function (mean ± SD)	86.88 ± 14.95	98.36 ± 18.43	< 0.001
Picture naming (mean ± SD)	9.36 ± 1.00	9.63 ± 0.67	0.081
Semantic fluency (mean ± SD)	17.59 ± 4.59	21.06 ± 5.80	< 0.001
Attention (mean ± SD)	101.40 ± 13.69	108.97 ± 14.17	< 0.001
Digit span (mean ± SD)	14.90 ± 1.68	14.66 ± 1.81	0.307
Coding test (mean ± SD)	44.88 ± 12.86	51.86 ± 12.56	< 0.001
Delayed memory (mean ± SD)	83.55 ± 14.75	93.89 ± 13.18	< 0.001
Story retelling (mean ± SD)	13.94 ± 4.35	15.48 ± 5.00	0.007
List recall (mean ± SD)	5.59 ± 2.45	6.57 ± 2.41	0.004
List recognition (mean ± SD)	19.17 ± 1.39	19.63 ± 0.80	0.005
Figure recall (mean ± SD)	12.15 ± 5.04	13.94 ± 4.49	0.019

BD, bipolar disorder; HC, healthy control; RBANS, Repeated Battery for the Assessment of Neuropsychological Status.

### 3.3 Correlation between CTQ scores and RBANS scores

We first analyzed the correlation between general factors such as age, gender, and RBANS scores. As shown in **Table 4**, years of education emerged as the strongest protective factor for cognitive function, demonstrating significant positive correlations with RBANS total score, immediate memory, language function, attention, and delayed memory. This relationship was consistent across both the BD and HC groups. In contrast, the number of episodes was identified as a major risk factor for cognitive function in BD patients, particularly showing a moderate negative correlation with immediate memory and attention. Age exhibited a negative correlation with RBANS total score and delayed memory in the HC group, whereas its impact in the BD group was less pronounced. Additionally, HAMD-17 scores showed no significant correlation with most cognitive functions.

**Table 4:** Correlations between general factors and cognitive function.

	Sex		Age		Married		Years of education		Positive family history of BD		Age at first onset		Totalduration of BD		Number of episodes		HAMD-17 total score		YMRS total score	
	BD	HC	BD	HC	BD	HC	BD	HC	BD	HC	BD	HC	BD	HC	BD	HC	BD	HC	BD	HC
RBANS total score	0.09	-0.02	0.03	-0.34^a^	0.00	0.30^a^	0.40^a^	0.52^a^	0.00	\	0.06	\	-0.02	\	-0.16	\	0.01	-0.06	-0.22^a^	0.13
Immediate memory	0.11	0.11	0.03	-0.32^a^	-0.08	0.30^a^	0.49^a^	0.43^a^	-0.01	\	0.18	\	-0.16	\	-0.25^a^	\	0.05	-0.13	-0.16	0.13
List learning	0.18	0.04	-0.12	-0.44^a^	0.03	0.40^a^	0.44^a^	0.44^a^	0.01	\	0.09	\	-0.22^a^	\	-0.30^a^	\	0.05	-0.07	-0.18	0.28^a^
Story recall	0.06	-0.05	-0.08	-0.44^a^	0.01	0.35^a^	0.41^a^	0.47^a^	-0.03	\	0.05	\	-0.14	\	-0.19	\	0.07	-0.15	-0.15	0.02
Visual span	0.04	-0.13	0.02	-0.20	0.08	0.14	0.19	0.30^a^	0.05	\	-0.04	\	0.12	\	-0.01	\	0.01	0.02	0.01	0.13
Graphic reproduction	-0.04	-0.15	-0.12	-0.35^a^	0.19	0.28^a^	0.06	0.33^a^	-0.07	\	-0.08	\	-0.04	\	-0.11	\	0.08	0.08	-0.11	0.05
Line orientation	0.03	-0.21^a^	-0.10	-0.28^a^	0.08	0.16	0.31^a^	0.28^a^	0.11	\	-0.16	\	0.13	\	0.02	\	-0.07	-0.06	0.03	0.28^a^
Language function	0.42^a^	0.04	0.15	-0.13	-0.10	0.10	0.30^a^	0.38^a^	0.05	\	0.16	\	0.11	\	-0.05	\	0.00	-0.01	-0.22^a^	0.03
Picture naming	0.21^a^	-0.18	0.14	-0.18	-0.12	0.08	0.15	0.36^a^	-0.04	\	0.11	\	0.10	\	0.01	\	-0.12	-0.06	-0.23^a^	0.13
Semantic fluency	0.34^a^	0.07	0.05	-0.17	-0.05	0.16	0.30^a^	0.39^a^	0.09	\	0.13	\	0.00	\	-0.09	\	0.05	0.02	-0.21^a^	0.03
Attention	0.01	0.00	0.04	-.23^a^	-0.04	0.21^a^	0.27^a^	0.35^a^	0.15	\	0.02	\	0.06	\	0.00	\	0.05	0.03	-0.13	0.06
Digit span	0.08	-0.18	-0.10	-0.41^a^	0.11	0.30^a^	0.23^a^	0.17	0.04	\	-0.12	\	0.07	\	0.14	\	-0.09	-0.04	-0.02	0.17
Coding test	0.06	-0.09	-0.12	-0.43^a^	-0.04	0.32^a^	0.27^a^	0.49^a^	0.17	\	-0.06	\	-0.05	\	-0.10	\	0.04	0.10	-0.14	0.05
Delayed memory	-0.06	-0.06	0.09	-0.07	-0.21^a^	0.05	0.34^a^	0.31^a^	-0.09	\	0.15	\	-0.09	\	-0.21^a^	\	0.06	0.02	-0.15	0.18
Story retelling	0.01	-0.10	-0.08	-0.45^a^	-0.02	0.36^a^	0.42^a^	0.43^a^	0.04	\	-0.03	\	-0.06	\	-0.13	\	0.05	-0.06	-0.11	0.17
List recall	0.11	-0.04	-0.09	-0.32^a^	-0.09	0.33^a^	0.23^a^	0.33^a^	0.04	\	0.04	\	-0.09	\	-0.19	\	0.02	-0.07	-0.17	0.22^a^
List recognition	-0.10	0.16	0.03	-0.13	-0.13	0.18	0.30^a^	0.29^a^	0.04	\	0.07	\	-0.11	\	-0.07	\	-0.10	-0.08	-0.19	-0.02
Figure recall	0.01	-0.21^a^	-0.07	-0.19	-0.07	0.19	0.11	0.34^a^	-0.02	\	-0.02		-0.02		-0.13		0.08	0.10	-0.03	-0.03

BD, bipolar disorder; HC, healthy control; RBANS, Repeatable Battery for the Assessment of Neuropsychological Status; HAMD-17, The 17-item Hamilton Depression scale; YMRS, The Young Mania Rating Scale. ^a^P<0.05.

The correlation between CTQ subscale scores and RBANS scores was analyzed across different groups. In BD patients, sexual abuse was negatively correlated with graphic reproduction (P < 0.05). In the HC group, emotional neglect was negatively correlated with delayed memory, while physical neglect was negatively correlated with RBANS total score, delayed memory, language function, and immediate memory (all P < 0.05). Detailed results of this analysis are presented in **Table 5**.

**Table 5:** Correlations between childhood trauma and cognitive function.

	Emotional abuse		Physical abuse		Sexual abuse		Emotional neglect		Physical neglect		Total CTQ score	
	BD With Childhood Trauma	BD Without Childhood Trauma	BD With Childhood Trauma	BD Without Childhood Trauma	BD With Childhood Trauma	BD Without Childhood Trauma	BD With Childhood Trauma	BD Without Childhood Trauma	BD With Childhood Trauma	BD Without Childhood Trauma	BD With Childhood Trauma	BD Without Childhood Trauma
RBANS total score	0.02	0.01	-0.13	-0.06	-0.03	-0.08	0.14	0.17	-0.16	-0.12	-0.04	-0.02
Immediate memory	0.00	-0.04	-0.03	-0.03	-0.02	-0.11	0.08	0.12	-0.20	-0.27	-0.04	-0.08
List learning	0.09	0.04	0.05	0.09	0.05	-0.11	0.04	0.04	-0.28	-0.36^a^	0.00	-0.09
Story recall	0.01	0.02	-0.08	-0.14	0.01	-0.09	0.06	0.10	-0.08	-0.16	-0.02	-0.08
Visual span	-0.09	-0.21	-0.20	-0.22	-0.15	-0.19	0.11	0.12	-0.10	-0.06	-0.12	-0.16
Graphic reproduction	0.04	-0.11	0.00	-0.08	-0.20	-0.25	-0.06	-0.07	0.04	0.05	-0.04	-0.14
Line orientation	-0.10	-0.05	-0.18	-0.07	0.10	0.07	0.03	0.14	-0.16	-0.20	-0.11	-0.04
Language function	-0.04	0.00	-0.11	-0.12	0.02	0.00	0.08	0.04	-0.14	-0.18	-0.06	-0.07
Picture naming	-0.09	-0.13	-0.12	-0.11	-0.01	-0.05	0.14	0.08	0.03	0.03	-0.02	-0.03
Semantic fluency	-0.05	0.00	-0.14	-0.10	-0.04	-0.02	-0.01	0.02	-0.26	-0.32^a^	-0.15	-0.15
Attention	-0.02	-0.01	-0.19	-0.03	-0.08	-0.08	0.07	0.08	-0.08	-0.11	-0.09	-0.08
Digit span	0.17	0.19	-0.04	0.07	0.09	-0.04	0.09	0.09	-0.10	-0.15	0.07	0.05
Coding test	-0.02	0.06	-0.13	0.04	-0.14	-0.14	-0.02	0.02	-0.15	-0.17	-0.13	-0.11
Delayed memory	-0.01	-0.07	-0.07	-0.13	0.04	-0.10	0.13	0.07	-0.07	-0.08	0.01	-0.10
Story retelling	-0.03	0.02	-0.02	-0.14	-0.02	-0.13	0.03	0.07	-0.15	-0.20	-0.05	-0.11
List recall	0.24	0.16	-0.05	-0.02	-0.03	-0.10	0.06	0.02	-0.21	-0.25	0.03	-0.08
List recognition	-0.12	0.01	-0.15	0.07	0.15	0.10	0.01	0.08	-0.17	-0.23	-0.10	0.01
Figure recall	0.01	-0.04	-0.02	-0.02	-0.14	-0.21	0.05	0.02	0.11	0.05	0.01	-0.11

	Emotional abuse		Physical abuse		Sexual abuse		Emotional neglect		Physical neglect		Total CTQ score	
	BD	HC	BD	HC	BD	HC	BD	HC	BD	HC	BD	HC
RBANS total score	-0.02	-0.04	-0.42	-0.06	-0.52	-0.17	0.21	-0.14	-0.34	-0.27^a^	-0.04	-0.24^a^
Immediate memory	-0.04	-0.15	-0.16	0.06	-0.51	-0.20	0.34	-0.17	-0.42	-0.31^a^	-0.02	-0.27^a^
List learning	0.08	-0.17	0.03	0.01	-0.08	-0.10	0.15	-0.09	-0.24	-0.15	0.01	-0.16
Story recall	-0.05	-0.16	-0.10	0.04	-0.12	-0.12	0.04	-0.16	-0.08	-0.28^a^	-0.02	-0.25^a^
Visual span	-0.21	0.05	-0.72	-0.16	-0.10	0.13	0.13	-0.11	-0.29	0.04	-0.10	-0.07
Graphic reproduction	-0.01	0.01	-0.02	-0.12	-0.16^a^	-0.16	-0.05	-0.02	-0.01	0.03	-0.01	-0.04
Line orientation	-0.01	0.02	-0.08	-0.05	0.12	0.15	0.06	-0.08	-0.01	0.10	0.00	0.02
Language function	-0.04	-0.06	-0.38	-0.00	-0.04	-0.02	0.02	-0.02	-0.42	-0.21^a^	-0.06	-0.12
Picture naming	-0.01	-0.22	-0.02	-0.07	-0.01	-0.16	0.01	0.05	-0.01	-0.31^a^	-0.00	-0.24^a^
Semantic fluency	-0.10	0.02	-0.20	-0.08	-0.21	-0.08	-0.09	-0.01	-0.28	-0.29^a^	-0.06	-0.16
Attention	-0.15	-0.03	-0.61	-0.08	-0.86	0.02	0.13	0.02	-0.14	-0.04	-0.07	-0.30
Digit span	0.05	0.04	-0.03	0.05	-0.00	-0.07	0.05	0.05	-0.03	-0.15	0.01	-0.03
Coding test	-0.23	-0.10	-0.43	-0.20	-1.14	0.04	0.10	-0.01	-0.06	0.03	-0.08	-0.05
Delayed memory	-0.10	-0.09	-0.31	-0.05	-0.14	-0.17	0.12	-0.21^a^	-0.31	-0.34^a^	-0.04	-0.31^a^
Story retelling	-0.06	-0.15	-0.03	-0.07	-0.10	-0.14	0.03	-0.07	-0.07	-0.26	0.00	-0.22^a^
List recall	0.07	-0.05	-0.06	-0.14	-0.10	-0.20	0.05	-0.21^a^	-0.11	-0.27^a^	-0.00	-0.30^a^
List recognition	-0.04	-0.02	-0.04	0.11	0.03	-0.01	-0.01	-0.08	-0.02	-0.16	-0.01	-0.09
Figure recall	-0.02	-0.09	-0.00	-0.12	-0.22	-0.15	0.04	-0.15	0.02	-0.25^a^	-0.00	-0.25^a^

CTQ, Childhood Trauma Questionnaire; BD, bipolar disorder; HC, healthy control; RBANS, Repeatable Battery for the Assessment of Neuropsychological Status; HAMD-17, The 17-item Hamilton Depression scale; YMRS, The Young Mania Rating Scale. ^a^P<0.05.

**Table 6** presents the analysis of the effects of different psychiatric medications on cognitive function in BD patients. The results indicate that the use of mood stabilizers showed a significant positive correlation with RBANS total score and language function, suggesting a potential protective effect of mood stabilizers on cognitive function. In contrast, valproate dosage was significantly negatively correlated with visual span. The use of antipsychotic medications appeared to be associated with a decline in attention function, whereas the impact of antidepressants on cognitive function was minimal, with most correlations being nonsignificant.

**Table 6:** The cognitive effects of psychiatric medications on BD.

	Whether mood stabilizers are used	Lithium carbonate dosage	Valproate dosage	Whether antipsychotics are used	Olanzapine equivalent dosage	Whether antidepressants are used	Fluoxetine equivalent dosage
RBANS total score	0.24^a^	0.03	-0.19	-0.12	-0.10	0.05	-0.01
Immediate memory	0.13	0.08	0.06	-0.06	-0.16	-0.08	-0.11
List learning	0.15	0.07	0.03	-0.02	-0.10	-0.12	-0.09
Story recall	0.17	0.03	0.01	-0.04	-0.12	-0.09	-0.17
Visual span	0.09	-0.08	-0.30^a^	-0.05	-0.17	0.11	-0.09
Graphic reproduction	0.03	-0.10	-0.22	-0.05	-0.12	0.04	0.17
Line orientation	0.19	0.01	-0.27	0.09	-0.19	-0.01	-0.23
Language function	0.23^a^	-0.04	-0.18	-0.10	-0.06	0.11	-0.01
Picture naming	0.12	0.10	-0.17	0.02	-0.14	0.09	0.12
Semantic fluency	0.25^a^	-0.11	-0.07	-0.12	-0.04	0.01	-0.09
Attention	0.10	-0.08	-0.25	-0.27^a^	0.07	0.04	0.23
Digit span	0.11	-0.23	-0.26	-0.22^a^	-0.09	-0.02	-0.02
Coding test	0.00	-0.06	-0.14	-0.24^a^	0.15	0.07	0.29
Delayed memory	0.17	-0.01	-0.14	0.03	0.04	0.06	-0.32
Story retelling	0.07	-0.05	-0.05	-0.09	-0.07	-0.13	-0.07
List recall	0.11	0.06	0.04	-0.01	0.08	-0.18	0.16
List recognition	0.10	-0.10	-0.03	0.03	0.14	0.03	-0.19
Figure recall	0.18	-0.03	-0.20	0.09	-0.01	0.06	0.01

CTQ, Childhood Trauma Questionnaire; RBANS, Repeatable Battery for the Assessment of Neuropsychological Status. ^a^P<0.05.

### 3.4 Regression analysis of RBANS scores

In the multivariate regression analysis of the five cognitive dimensions of RBANS and the five subfactors of CTQ, emotional abuse was significantly associated with immediate memory function in the BD group. Further multivariate regression analysis of RBANS total scores revealed that, in the BD group, years of education were significantly positively correlated with RBANS total scores, suggesting a potential positive impact on cognitive function. In contrast, the number of episodes and valproate dosage were significantly negatively correlated with RBANS total scores, indicating these factors might have adverse effects on cognitive function. In the HC group, years of education were also significantly positively correlated with RBANS total scores, while CTQ total scores were negatively correlated with RBANS total scores. Other variables, such as gender, age, marital status, and the use of antipsychotic medications, showed no significant effect on RBANS total scores. Detailed results of this analysis are presented in **Table 7**.

**Table 7:** Regression analysis of factors affecting cognitive function.

Multivariate regression analysis	Variant	Type III Sum of Squares		*F*-value		*P*-value	
		BD	HC	BD	HC	BD	HC
Emotional abuse	Immediate memory	5,712.47	1,487.61	2.21	0.60	**0.04**	0.75
	Visual span	2,555.61	1,535.10	0.76	0.85	0.67	0.55
	Language function	1,611.82	2,587.67	0.89	1.33	0.56	0.26
	Attention	1,537.49	1,724.55	0.63	1.34	0.79	0.25
	Delayed memory	4,562.95	800.19	2.00	0.63	0.06	0.73
Physical abuse	Immediate memory	1,287.87	1,806.36	0.91	1.03	0.50	0.41
	Visual span	263.74	1,049.88	0.14	0.81	0.99	0.55
	Language function	634.91	2,656.05	0.64	1.91	0.70	0.11
	Attention	678.72	716.38	0.51	0.78	0.80	0.57
	Delayed memory	1,918.09	742.69	1.54	0.81	0.19	0.55
Sexual abuse	Immediate memory	1,115.53	452.32	0.79	0.32	0.58	0.86
	Visual span	867.37	295.93	0.47	0.29	0.82	0.89
	Language function	1,215.14	2,503.02	1.23	2.25	0.32	0.08
	Attention	367.55	1,819.78	0.27	2.47	0.95	0.06
	Delayed memory	1,749.59	770.82	1.41	1.05	0.24	0.39
Emotional neglect	Immediate memory	3,618.02	1,821.79	1.10	0.43	0.39	0.94
	Visual span	3,024.73	3,571.62	0.71	1.15	0.75	0.34
	Language function	3,780.76	3,392.53	1.64	1.02	0.12	0.45
	Attention	3,296.52	2,355.14	1.05	1.06	0.43	0.41
	Delayed memory	1,770.39	1,177.91	0.61	0.54	0.84	0.88
Physical neglect	Immediate memory	3,229.64	2,016.74	1.38	0.64	0.23	0.76
	Visual span	1,786.63	2,682.03	0.59	1.15	0.81	0.34
	Language function	1,432.99	2,208.24	0.87	0.88	0.57	0.55
	Attention	618.90	1,877.41	0.28	1.13	0.98	0.36
	Delayed memory	2,272.24	2,116.41	1.10	1.29	0.39	0.27

Simple Linear Regression	Variant	B-value		t-value		*P*-value	
		BD	HC	BD	HC	BD	HC
RBANS total score	Sex	0.11	0.01	1.14	0.06	0.26	0.96
	Age	-0.03	-0.19	-0.28	-1.48	0.78	0.14
	Married	0.03	-0.05	0.25	-0.36	0.80	0.72
	Years of education	0.40	0.45	4.20	4.46	**0.00**	**0.00**
	YMRS total score	0.03	0.03	0.31	0.35	0.75	0.73
	Total duration of BD	0.05	/	0.40	/	0.69	/
	Number of episodes	-0.24	/	-2.07	/	**0.04**	/
	Whether mood stabilizers are used	0.30	/	2.47	/	**0.02**	/
	Valproate dosage	-0.26	/	-2.35	/	**0.02**	/
	Whether antipsychotics are used	-0.00	/	-0.03	/	0.98	/
	CTQ	0.04	-0.20	0.42	-2.16	0.67	**0.03**

BD, bipolar disorder; HC, healthy control; CTQ, Childhood Trauma Questionnaire.

Bold values: P < 0.05.

## 4 Discussion

This study investigated the relationship between different childhood trauma experiences and cognitive impairments across various domains in patients with bipolar disorder (BD). Through the analysis of correlations between CTQ scores and RBANS scores in BD patients, as well as multivariate regression analysis of RBANS scores, we found that emotional abuse was negatively correlated with immediate memory ability. Additionally, the study revealed that years of education were positively correlated with RBANS total scores and several cognitive abilities, suggesting that longer durations of education may be associated with less severe cognitive impairments in BD patients.

The findings of this study revealed a high incidence of childhood trauma among patients with BD, with the most common forms being physical neglect, emotional neglect, sexual abuse, emotional abuse, and physical abuse. The results of this study also indicated that the proportion of BD patients with a history of emotional neglect is significantly higher than that among the HC population. The existing research indicates that patients with schizophrenia, BD, and obsessive-compulsive disorder have higher levels of childhood trauma compared with the normal population. Noteworthy distinctions are evident, particularly in the domains of general trauma, sexual trauma, and emotional abuse, when comparing psychological disorder groups to HC groups (23). In a study of psychiatric outpatients in Korea, the most common forms of childhood trauma are sexual abuse, emotional abuse, physical abuse, physical neglect, and emotional neglect, in that order, consistent with trends seen in other populations and patient groups (24).

The findings of the present study align with prior research (10) and indicate that childhood trauma significantly affects the cognitive function of BD patients. According to Ehrlich et al. (25), adults diagnosed with BD who have encountered elevated levels of childhood trauma demonstrate diminished memory function compared to adult BD patients with lower levels of childhood trauma. The study by Dauvermann and Donohoe (26) suggests that BD patients who experienced significant childhood emotional neglect perform worse on recognizing the emotion of anger compared with BD patients without such experiences. Hsieh et al. (13) observed a negative correlation between the degree of physical neglect and executive function performance in BD patients.

However, the impact of childhood trauma on cognitive function differs among various patient cohorts. For example, findings from the study by Popovic et al. (27) illustrate an association between childhood trauma and impaired working memory, attention, executive function, and verbal learning in patients diagnosed with schizophrenia. Increased occurrences of childhood trauma in these patients are associated with heightened attenuated positive symptoms, depressive symptoms, general symptoms, reduced overall functioning, and impaired cognitive performance on tasks related to visual episodic memory and executive functions. Zhang (28) showed that multiple forms of childhood trauma, such as emotional neglect, sexual abuse, emotional abuse, and physical abuse, are moderately associated with panic disorder. A tendency toward dissociation may emerge when patients try to integrate adverse experiences into their cognitive schema. The study by Liu and colleagues (29) confirmed that exposure to emotional abuse and emotional neglect substantially raises the likelihood of experiencing a first episode of depression. Use of childhood trauma as a predictor for the initial episode of depression can enhance the accuracy of treatment approaches.

The findings of this study highlight a significant association between emotional abuse and impairments in immediate memory function, aligning with previous research that has suggested a link between emotional maltreatment and deficits in spatial working memory. Emotional abuse may contribute to memory deficits in adulthood, which, in turn, could serve as a risk factor for the development of psychopathology (30). Previous studies have explored the mechanisms underlying this relationship, suggesting that childhood emotional maltreatment has a detrimental effect on the morphology of the medial prefrontal cortex (mPFC) (31). The mPFC plays a critical role in the regulation of cognitive function and emotional memory and is closely interconnected with the limbic system. Individuals who have experienced childhood emotional maltreatment exhibit reduced functional activity in the mPFC, a key brain region associated with emotion and memory. This diminished activity in the mPFC may represent a neural mechanism underlying long-term memory impairments. These findings underscore the importance of addressing childhood emotional maltreatment as a potential contributor to memory dysfunction and the broader risk of psychopathology, emphasizing the need for early interventions to mitigate its long-term impact on brain function and mental health.

The precise mechanisms by which childhood trauma influences cognitive function remain incompletely understood. Pertinent research suggests that the prefrontal cortex (32), acknowledged as one of the regions consistently affected by childhood trauma, mediates executive function and working memory (33). Stress is associated with temporary activation of the hypothalamic-pituitary-adrenal (HPA) axis, leading to increased secretion of hormones, including cortisol. In the context of acute stress, this is a normal and short-term response. However, persistent or recurrent exposure to stress, as occurs in cases of abuse, is linked to enduring alterations in activation of the HPA axis and its pathways (34). When stress occurs during sensitive developmental periods, these alterations are more likely to occur, and prolonged periods of high cortisol levels, particularly during childhood, might result in diminished hippocampal volume, thereby constraining the processes of learning and memory formation (35).

Imaging studies in BD patients (36) showed that the degree of physical neglect is negatively correlated with the functional connectivity between the left caudate nucleus and the frontal cortex (37). The degree of physical neglect is also negatively correlated with executive function. These findings suggest that childhood trauma may impair cognitive function by influencing the functional connectivity of the frontal cortex–caudate circuit. Other studies have proposed that childhood trauma may induce alterations in the structure, function, and connectivity of critical brain regions associated with cognition, including the prefrontal cortex (38), hippocampus, and amygdala (39). Moreover, changes in the integrity of white matter fiber tracts, particularly in the corpus callosum, have been observed in patients with a history of childhood trauma (32).

## 5 Limitations

This study has several limitations. First, data on adverse childhood events were collected retrospectively, which introduces inherent weaknesses associated with retrospective reporting designs. Second, the tools used in this study were relatively simple, and the single-center design raises the potential for selection bias. Third, this study aimed to investigate the relationship between childhood trauma and cognitive function in bipolar disorder (BD). While manic episodes were excluded from the study, some patients included were in a depressive episode rather than in remission. Depressive episodes themselves may impact cognitive function, and this was not accounted for in the analysis. Finally, the healthy control group was not strictly matched to the experimental group in terms of factors such as age and years of education, which may have contributed to a higher number of positive results in the correlation analysis for the control group compared to the experimental group. Additionally, in the correlation analysis, multiple factors were associated with various cognitive functions. Due to the large number of covariates, we did not include all covariates in the multivariate regression analysis, which may have influenced the results of the study.

## 6 Conclusion

The results of this study confirm that the incidence rates of various forms of childhood trauma are higher among patients with bipolar disorder (BD) compared to the general population. These childhood trauma experiences are associated with cognitive impairment in BD patients, particularly in the memory domain. Furthermore, as the severity of childhood trauma experienced by BD patients increases, the likelihood of cognitive impairment also rises. Cognitive function in BD is not only linked to trauma but also to factors such as years of education and the number of episodes, as well as medications taken by BD patients, including mood stabilizers and antipsychotics. The study identified emotional abuse as a predictor of impaired immediate memory, with years of education serving as a protective factor for cognitive function, while valproate dosage emerged as a risk factor. Future research should focus on exploring the relationships between the types and severities of childhood trauma and the clinical characteristics of BD patients, the impact of neglect-related trauma, and the underlying mechanisms connecting childhood trauma to cognitive impairment. Additionally, developing targeted interventions to reduce the incidence of BD and improve patient outcomes is essential. Such studies will provide critical insights into the complexity of BD and support the development of more effective treatment strategies.

## Data Availability

The raw data supporting the conclusions of this article will be made available by the authors, without undue reservation.
